# Precision acupuncture for chemotherapy-induced peripheral neuropathy: multitarget mechanisms and integrated clinical translation framework

**DOI:** 10.3389/fneur.2026.1855553

**Published:** 2026-09-10

**Authors:** Shuo Ding, Pengfei Qi, Hongbo Zhang, Mengdi Yu, Jinsong Dang, Hongna Yin

**Affiliations:** 1 Heilongjiang University of Chinese Medicine, Harbin, China; 2 The Second Affiliated Hospital of Heilongjiang University of Chinese Medicine, Harbin, China

**Keywords:** acupuncture, chemotherapy-induced peripheral neuropathy, pathogenesis, precision medicine, treatment

## Abstract

Chemotherapy-induced peripheral neuropathy (CIPN), a debilitating dose-limiting toxicity affecting 20–85% of cancer patients, lacks disease-modifying therapies. This review synthesizes evidence supporting acupuncture as a potential multi-target strategy against CIPN. Direct preclinical CIPN studies suggest that electroacupuncture (EA) may modulate selected neuroinflammatory and oxidative pathways, including TLR4/NF-κB signaling, TRPV1 upregulation, and spinal glial activation; proposed effects on Nav1.7–1.9, PGC-1α/AMPK, or HDAC6-related microtubule stabilization remain unconfirmed. Clinically, sham-controlled trials suggest that manual acupuncture and EA may alleviate pain, sensory symptoms, and quality-of-life impairment. High response rates for auricular acupuncture derive from an uncontrolled case series, and ALTENS findings are preliminary. The MC5-A Calmare device delivers Scrambler therapy and is distinct from ALTENS and acupuncture. Combination studies suggest possible additive benefit rather than established synergy. Safety profiles are generally favorable. However, clinical translation is impeded by methodological heterogeneity, subjective “De Qi” assessment, and inadequate patient stratification. Future research should focus on trials with lab markers and cost analyses to determine how well acupuncture helps people complete their treatment.

## 1 Introduction

Chemotherapy-induced peripheral neuropathy (CIPN) is a common problem that can limit treatment (1). It occurs when drugs such as platinum compounds, taxanes, and vinca alkaloids damage nerves—primarily those that control sensation, movement, and automatic bodily functions (2). Patients usually notice numbness, tingling, pain, or odd sensations in their hands and feet. These symptoms often appear in a symmetrical, glove-and-stocking pattern and usually start early in chemotherapy. Some people improve after treatment ends, but many have ongoing symptoms that make daily life and quality of life worse (3, 4).

The clinical and epidemiological burden of CIPN is considerable, with its incidence exhibiting substantial heterogeneity across patient populations and chemotherapeutic regimens. Studies indicate that the overall prevalence of CIPN among chemotherapy patients is approximately 48%, while the incidence rate reaches 68.1% within 1 month after chemotherapy completion; the cumulative prevalence persists at 28.7% 1 year post-chemotherapy (5, 6). The chance of getting CIPN ranges from 20 to 85%, depending on the chemotherapy drug. Paclitaxel is linked to some of the highest rates (7, 8). The risk increases with higher total doses—more than 300 mg/m^2^ of oxaliplatin or taxanes increases the risk of nerve problems (9). This high prevalence underscores CIPN’s role as a major dose-limiting toxicity, often necessitating chemotherapy dose reduction or discontinuation that directly compromises oncological outcomes. Importantly, about half of patients report chronic symptoms that profoundly impair quality of life, with severe CIPN frequently leading to treatment cessation (10, 11).

Currently, no disease-modifying therapy is approved for CIPN. Clinical management primarily relies on symptomatic relief using drugs such as certain antidepressants and anticonvulsants. The 2020 American Society of Clinical Oncology guideline identifies duloxetine as the only agent with appropriate evidence for established painful CIPN, while noting that its average benefit is limited (12). Nevertheless, the efficacy of this pharmacologic approach is limited. The treatment strategies remain limited to symptomatic control rather than mechanistic intervention. Because we do not fully understand how CIPN develops (7, 11), it’s hard to design targeted drugs. As a result, these strategies yield suboptimal outcomes characterized by variable patient response, high rates of symptom recurrence upon drug withdrawal, and frequent adverse effects (e.g., dizziness, somnolence, gastrointestinal disturbances) (1). Non-drug treatments like acupuncture also have mixed results, since studies often find little difference between real and fake treatments (13, 14). All of this makes it hard to know what truly works and what does not. Taken together, these challenges show there’s a real need for new treatments that target the causes of CIPN and are easier for patients to handle.

Traditional Chinese medicine (TCM), with its characteristic multi-targeted approach, offers a diverse range of interventions for CIPN, including acupuncture, moxibustion, Chinese herbal medicine (both internal and external applications), and acupressure. Of these, acupuncture has been extensively studied and shows promise in clinical applications. The therapeutic rationale is from a dual theoretical framework: the TCM principle of “unblocking collaterals to alleviate pain”, historically applied for pain and neurological disorders, and modern neuromodulation theory involving multi-target mechanisms, such as anti-neuroinflammation, ion channel regulation, and neuroprotection/recovery (13). Systematic reviews and meta-analyses suggest that acupuncture and related therapies, theoretically aligned with these principles, may not only help alleviate sensory abnormalities and improve muscle strength in patients with CIPN but also enhance their overall quality of life (15). Some benefits, such as pain reduction, may be observed as early as the second week of treatment (16). Compared with some Western treatments that primarily address symptoms and may have side effects or limited efficacy (15), acupuncture appears safe in studies, with few reported adverse effects (17, 18). Acupuncture-related interventions investigated for CIPN include manual acupuncture (MA), electroacupuncture (EA), auricular acupuncture, moxibustion, and transcutaneous electrical acupoint stimulation (TEAS). Among these modalities, MA and EA are currently supported by the greatest amount of clinical evidence, whereas auricular acupuncture and TEAS provide minimally invasive alternatives with encouraging preliminary efficacy in symptom control and functional recovery.

Based on current clinical needs and accumulating evidence, this paper aims to review the recent domestic and international literature to summarize the pathogenesis of CIPN and critically assess the efficacy and potential mechanisms of acupuncture-related therapies for its prevention and treatment. In particular, this review contributes novel insights through: (1) a systematic synthesis of the latest clinical evidence regarding acupuncture’s efficacy, safety profile, and comparative effectiveness relative to other treatments for CIPN; (2) an in-depth exploration of the potential multi-target molecular mechanisms through which acupuncture may ameliorate CIPN, with particular focus on anti-neuroinflammation, ion channel regulation, and neuroprotection; and (3) the proposal of a ‘precision acupuncture’ clinical translation framework designed to guide future research and clinical practice. By pulling together what is already known and highlighting what remains unclear, we hope to lay the groundwork for future research and the practical use of acupuncture for CIPN.

## 2 Pathogenesis of CIPN: targeted pathways of acupuncture intervention

**Figure 1:**
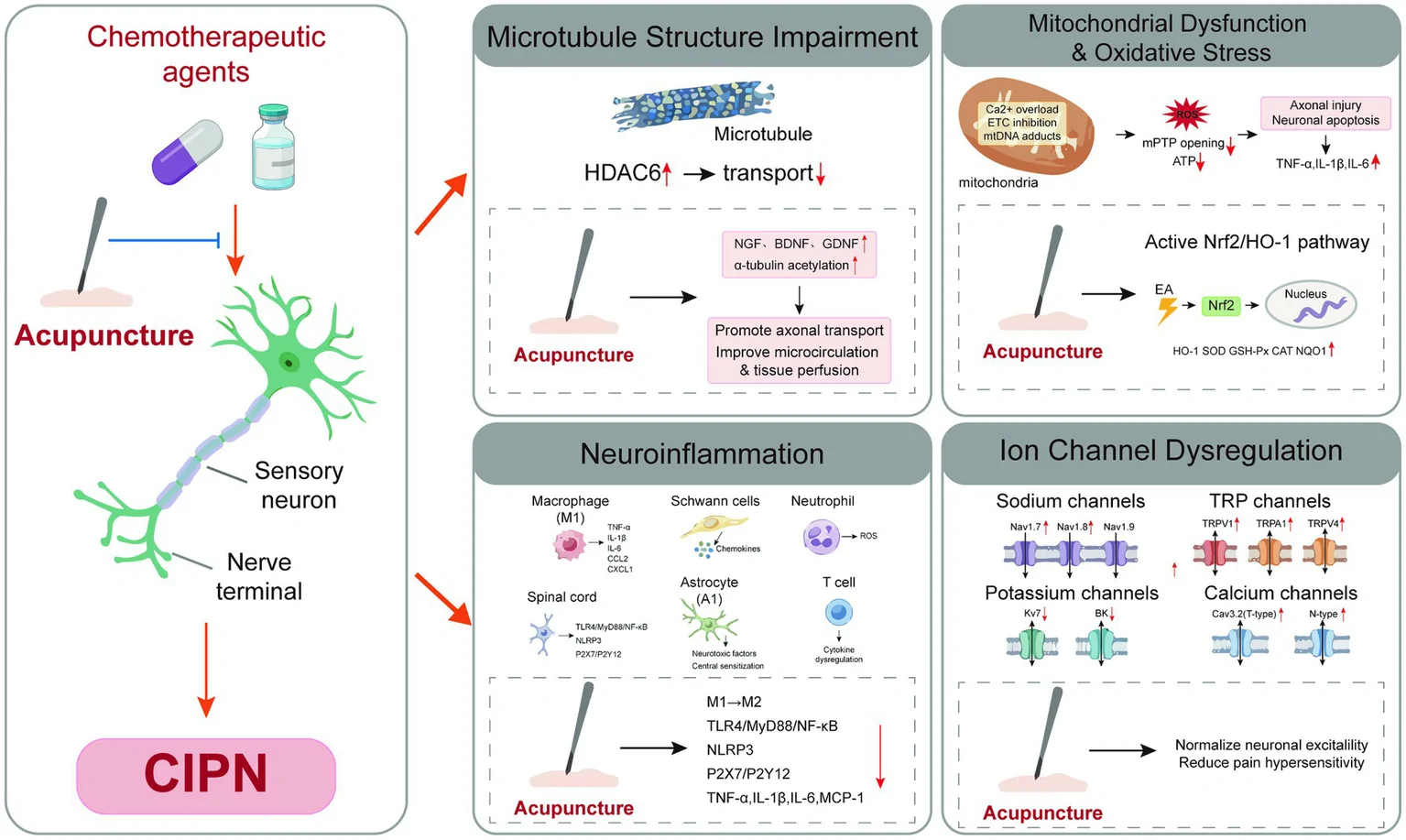
Acupuncture’s multitarget modulation of CIPN pathways.

The core pathogenic mechanisms, including alterations in microtubule structure, mitochondria and oxidative stress, neuroinflammation, and abnormal expression of ion channels, are summarized in Figure 1.

### 2.1 Alterations in microtubule structure

Microtubules are major cytoskeletal structures composed of α/β-tubulin heterodimers that maintain axonal architecture and support intracellular transport required for neuronal function. Findings from non-chemotherapy peripheral nerve injury models provide indirect biological plausibility that acupuncture may support axonal repair (19), but direct evidence that acupuncture preserves microtubules or microtubule-dependent transport in CIPN is currently lacking.

Taxanes excessively stabilize microtubules, whereas vinca alkaloids inhibit tubulin polymerization. Despite these opposite primary actions, both drug classes disrupt the physiological rhythms of axonal transport, leading to disruptions in the transport of organelles such as mitochondria, messenger RNA (mRNA), and proteins. This leads to the accumulation or depletion of substances within nerve fibers, resulting in axonal degeneration and ultimately triggering CIPN (20). Research indicates that histone deacetylase 6 (HDAC6) disrupts microtubule stability and impedes α-tubulin-mediated mitochondrial transport *in vitro*, while *in vivo* it promotes the onset and progression of CIPN (21). Treatment with highly selective HDAC6 inhibitors effectively reverses cisplatin-induced nerve damage and peripheral neuropathy symptoms while promoting the repair of intraepidermal nerve fibers. Additionally, mitochondrial dysfunction readily leads to an insufficient supply of adenosine triphosphate (ATP), which is required for axonal transport, thereby impairing axonal transport. This speeds up nerve damage and makes CIPN worse (22).

Acupuncture may preserve microtubule integrity and facilitate axonal transport, thereby promoting peripheral nerve repair. HDAC6, a major tubulin deacetylase, disrupts microtubule stability and impairs axonal transport when overactive; its inhibition has been shown to restore acetylation, improve mitochondrial trafficking, and reverse CIPN symptoms, underscoring microtubule stabilization as a therapeutic strategy. Direct evidence for acupuncture-mediated HDAC6 regulation in CIPN is still limited (23, 24). Studies in peripheral nerve injury models indicate that electroacupuncture promotes axonal regeneration and neural remodeling by enhancing the retrograde transport of neurotrophic factors such as NGF, BDNF, and GDNF, together with cytoskeletal remodeling. These regenerative effects may indirectly facilitate microtubule-dependent axonal transport and mitochondrial delivery to distal axons, although direct evidence for this mechanism remains limited. In addition, acupuncture has been shown to improve local microcirculation and tissue perfusion, which may provide the metabolic support required for microtubule-dependent repair (19).

### 2.2 Mitochondria and oxidative stress

Chemotherapy drugs such as taxanes and platinum compounds disrupt mitochondrial function, which is a key cause of chronic, distal, and bilateral peripheral neuropathy, often with neuropathic pain (22). Evidence explores the links between CIPN and mitochondrial damage in primary sensory neurons, which can worsen nerve pain (25). Paclitaxel disrupts intracellular calcium homeostasis, impairs mitochondrial respiratory chain activity, reduces ATP synthesis, and promotes excessive ROS production, ultimately leading to axonal degeneration (26, 27). This stress also activates the immune system, increasing inflammatory molecules such as TNF-α and IL-1β, and worsening CIPN (28–30). Animal studies have highlighted key features of paclitaxel-induced mitochondrial dysfunction in dorsal root ganglia (DRG) (30), and further research shows these energy changes in DRG neurons are linked to the onset and persistence of paclitaxel-induced pain.

Platinum-based chemotherapy drugs, such as oxaliplatin and cisplatin, can harm nerves by binding to mitochondrial DNA (mtDNA) within cells (31–33). The repair mechanisms allow these adducts to accumulate, disrupting mtDNA replication and transcription and ultimately impairing the synthesis of essential proteins, including those that constitute the respiratory chain complexes (26). As a result, the mitochondrial electron transport chain cannot function properly, leading to impaired cellular energy metabolism. Excess reactive oxygen species (ROS) add further stress, causing greater nerve damage and sometimes triggering cell death in sensitive cells (32, 34–36). Importantly, the mechanisms underlying oxidative stress-mediated hyperalgesia differ depending on the drug: cisplatin mostly affects nerves outside the spinal cord and sensitizes pain receptors, while paclitaxel increases spinal cord stress and prolongs abnormal pain signals from Aβ fibers (37, 38). When mitochondria are damaged, they also lose their membrane potential, further reducing energy production (29). Studies show that chemotherapy-related stress also disrupts how mitochondria split, fuse, and are formed. This hurts energy output, calcium balance, and overall mitochondrial health (26). These problems combine to drive the development and worsening of CIPN (26, 27, 29, 37).

Experimental studies indicate that electroacupuncture may preserve mitochondrial membrane potential, restore ATP generation, and attenuate mitochondrial swelling, thereby improving neuronal energy metabolism and reducing axonal degeneration. However, most such studies have been performed in peripheral nerve injury or central nervous system disease models rather than CIPN specifically (39). In a rat model of paclitaxel-induced neuropathic pain, electroacupuncture enhanced Nrf2-antioxidant response element signaling and superoxide dismutase levels, reduced oxidative products, and attenuated hypersensitivity (40). This finding supports Nrf2-related antioxidant modulation but does not establish restoration of mitochondrial biogenesis or clinical efficacy in humans.

### 2.3 Neuroinflammation

CIPN involves abnormal neuroinflammatory responses, in which cytokine signaling pathways make sensory neurons more sensitive, a main factor in CIPN development (41). The TLR4/NF-κB axis functions as a central regulatory pathway. Chemotherapeutic agents activate TLR4 on microglial cells, initiating MyD88-dependent recruitment of IRAK4 and TRAF6. This signaling cascade results in IκB kinase (IKK)-mediated degradation of IκBα, which releases NF-κB dimers (p65/p50) that translocate to the nucleus and promote transcription of proinflammatory cytokines, including TNF-α, IL-1β, and IL-6. These cytokines contribute to neuronal damage and further amplify inflammation by activating microglia, thereby establishing a self-perpetuating cycle of injury (42–44). Recent evidence indicates that neuroinflammation in CIPN is orchestrated by coordinated interactions among both resident and infiltrating immune cells. Following chemotherapy, macrophages infiltrate peripheral nerves and dorsal root ganglia (DRG), where M1-polarized macrophages release TNF-α, IL-1β, IL-6, CCL2, and CXCL1, thereby amplifying neuronal injury and peripheral sensitization. Schwann cells also participate in this inflammatory cascade by producing chemokines that recruit circulating monocytes and macrophages while simultaneously releasing inflammatory mediators that impair axonal regeneration. During the early phase of chemotherapy-induced injury, neutrophils further aggravate oxidative stress through excessive reactive oxygen species production, whereas adaptive immune cells, particularly T lymphocytes, contribute to persistent neuroimmune dysregulation by modulating cytokine production and glial activation. Within the spinal cord, activated microglia and astrocytes establish a self-perpetuating inflammatory loop that maintains central sensitization and chronic neuropathic pain (45). Meanwhile, NLRP3 inflammasome is a key part of nerve inflammation. When chemotherapy damages mitochondria, it can trigger NLRP3 oligomerization, leading to caspase-1-dependent maturation of IL-1β and IL-18 (46, 47), further disrupting neuronal homeostasis. Notably, chemokines (e.g., CCL2-5) released by active microglia recruit macrophages and contribute to nerve pain (48, 49). This also strengthens how glial cells communicate, especially by pushing astrocytes to become the harmful A1 type (50).

Acupuncture may modulate neuroimmune interactions at multiple levels, but much of the supporting literature is derived from non-CIPN models (45). Direct preclinical CIPN evidence is more specific. In paclitaxel-treated rats, electroacupuncture inhibited spinal glial activation and TLR4/NF-κB signaling (51). In cisplatin-treated mice, preventive electroacupuncture reduced pro-inflammatory microglial activity through neuronal GRK2- and miR-124-dependent mechanisms (52, 53). In another paclitaxel model, electroacupuncture reduced CCL2/CCR2-mediated recruitment of M1-like macrophages without increasing M2-like macrophages (54). These data support selected, model-specific neuroimmune effects.

Preclinical studies suggest that acupuncture may regulate innate immune cells, glial activation, inflammatory signaling cascades, and neuroimmune communication. Such multi-level regulation may explain its potential effects in CIPN, but these model-specific pathway changes have not been established as mediators of clinical benefit.

### 2.4 Ion channels

CIPN primarily results from abnormal expression of voltage-gated ion channels, especially sodium channels (Nav). Chemotherapeutic agents upregulate Nav1.7, Nav1.8, and Nav1.9 subtypes in sensory neurons, increasing action potential generation and neuronal hyperexcitability (55–57). Extra sodium currents (INaP) worsen CIPN by keeping nerve cells depolarized and lowering the threshold for firing, increasing sensitivity and pain (58, 59). In addition, sodium channel β-subunits (SCN1B-SCN4B), once considered only accessory proteins, have non-conductive roles critical to pathology. They regulate channel localization, neuro-glial interactions, and adhesion. Genetic variants in β-subunits destabilize the Nav complex and increase susceptibility to neuropathic pain, highlighting new therapeutic targets (60–62).

Dysregulation of potassium and calcium channels exacerbates CIPN. Chemotherapeutics, such as oxaliplatin/paclitaxel, profoundly suppress Kv7 subtypes in dorsal root ganglion (DRG) neurons, reducing repolarization capacity and increasing excitability (63). This problem also affects BK channels, which help control the nerve cell’s membrane potential (64, 65). In models of CIPN caused by taxanes, the expression of T-type calcium channels (Cav3.2) increases. Cav3.2 channels become even more sensitive when BDNF–TrkB signaling is increased, which means they open more readily and let in more calcium, increasing the likelihood that nerves will fire (66). Pharmacological inhibition of T-type, N-type, or α2δ calcium channels reverses mechanical/thermal hyperalgesia in CIPN models (67), confirming their therapeutic relevance.

In addition to voltage-gated channels, transient receptor potential (TRP) channels play a central role as multimodal transducers in the development of CIPN pain. Chemotherapeutic agents such as platinum and taxane compounds activate TRPV1 and TRPA1 channels on sensory neurons, which leads to increased thermal and mechanical hypersensitivity (38, 68, 69). TRPV1 primarily detects heat and inflammatory mediators, while TRPA1 is sensitive to oxidative stress and cold stimuli. TRPV4 contributes to the transduction of mechanical signals. Together, these channels mediate the allodynia and hyperalgesia observed following chemotherapy (70). Interventions that target these pathways, such as the use of antioxidants, such as aminobutyronitrile to reduce oxidative stress (68), TRPV1 antagonists, including capsaicin to alleviate paclitaxel-induced allodynia (71), and TRPA1 inhibitors to reverse symptoms in models of paclitaxel and bortezomib exposure (72), have demonstrated efficacy in mitigating TRP-mediated pain.

Acupuncture may influence ion-channel-related signaling in CIPN; direct evidence is currently strongest for TRPV1. In paclitaxel-treated rats, electroacupuncture reduced TLR4 signaling and TRPV1 upregulation in sensory neurons (73). Claims that acupuncture downregulates Nav1.7–Nav1.9, reduces persistent sodium current, restores Kv/BK channels, or attenuates Cav3.2 sensitization in CIPN should be treated as hypotheses for future mechanistic studies.

### 2.5 Integrated molecular mechanisms of acupuncture intervention in CIPN

Accumulating experimental and clinical evidence suggests that acupuncture may alleviate chemotherapy-induced peripheral neuropathy through coordinated regulation of multiple interconnected molecular and cellular pathways. Because the pathological mechanisms of CIPN—including microtubule disruption, mitochondrial dysfunction, oxidative stress, neuroinflammation, ion channel dysregulation, and neuroimmune imbalance—are highly interdependent, acupuncture appears to exert a systems-level regulatory effect capable of modulating several key pathogenic cascades. However, direct CIPN evidence does not yet establish simultaneous modulation of all of these pathways; this proposed multi-target characteristic should therefore be interpreted as a working model rather than an established acupuncture mechanism.

At the structural level, acupuncture may preserve neuronal cytoskeletal integrity by maintaining microtubule stability and facilitating axonal transport; however, this remains hypothetical in CIPN. Future mechanistic studies should clarify whether acupuncture directly modulates microtubule acetylation pathways. Mitochondrial protection represents another potential mechanism through which acupuncture may alleviate CIPN. Although most mechanistic evidence originates from peripheral nerve injury and central nervous system disease models, these biological processes are highly relevant to the oxidative stress observed in CIPN. Neuroimmune regulation may constitute another therapeutic mechanism of acupuncture. Chemotherapy-induced activation of macrophages, Schwann cells, microglia, astrocytes, neutrophils, and T lymphocytes establishes a persistent inflammatory microenvironment within peripheral nerves, dorsal root ganglia, and the spinal cord. Acupuncture may also modulate neuronal excitability through ion-channel-related signaling; direct CIPN evidence is strongest for TRPV1, whereas regulation of Nav/Kv/Cav channels remains unconfirmed. These local effects may be integrated through systemic neuroimmune communication.

Collectively, current evidence supports acupuncture as a systems-level intervention capable of simultaneously regulating neuronal, immune, metabolic, and neuroendocrine networks involved in CIPN. Nevertheless, most mechanistic evidence has been derived from animal models or neurological disorders other than CIPN. Future studies integrating transcriptomics, proteomics, metabolomics, single-cell sequencing, spatial transcriptomics, and neuroimaging with well-designed clinical trials will be essential to establish causal relationships between acupuncture-induced molecular regulation and clinical improvement, thereby facilitating biomarker-guided precision acupuncture for CIPN.

## 3 Clinical evidence on acupuncture for the treatment of CIPN

The clinical evidence for acupuncture in CIPN is organized by therapeutic approach, as presented in Table 1. We summarized key trial data across five principal techniques: manual acupuncture (MA), electroacupuncture (EA), moxibustion, combination therapies, and specialized techniques. This section highlights comparative efficacy metrics, treatment protocols, safety profiles, and limitations, providing a consolidated reference for selecting evidence-based modalities.

**Table 1:** Clinical evidence and therapeutic characteristics of acupuncture-based interventions for chemotherapy-induced peripheral neuropathy.

Therapy	Representative cancer population	Representative acupoints	Treatment protocol	Mechanism	Primary outcomes	Advantages	Disadvantages
Manual acupuncture	Breast cancer survivors receiving taxane chemotherapy	LI4, LI11, ST36, SP6, LR3, Baxie (EX-UE9), Bafeng (EX-LE10), individualized according to symptom distribution.	1–2 sessions/week; 20–30 min/session; total 8–10 weeks.	Stimulates nerve repair; modulates pain pathways via de-qi sensation	↓ Pain (VAS/NRS), ↑ sensory function, ↑ QoL	Non-pharmacologic; minimal side effects	Needle aversion; session frequency
EA	Colorectal cancer receiving oxaliplatin; breast cancer receiving taxanes.	ST36, GB34, LI4, SP6 (bilateral); additional individualized points according to symptoms.	2 Hz (or 2/100 Hz); 30 min/session; once or twice weekly; 10–12 weeks.	Suppresses TRPV1/TLR4/NLRP3; modulates Na + channels (Nav1.7–1.9); enhances endogenous opioids; preserves mitochondrial function	↑ FACT/GOG-Ntx scores; ↓ NRS pain; improved physical function.	Multi-target neuroprotection; >90% patient compliance; minimal adverse events (minor hematomas: <1%)	Requires specialized equipment/training; limited long-term efficacy data
Moxibustion	Mixed cancer populations receiving platinum- or taxane-based chemotherapy.	ST36, SP6, CV4, local painful areas	20–30 min/session; 2–3 sessions/week.	Local heat enhances blood flow, modulates nerve activity, and may suppress neuroinflammation through thermal bioeffects.	Reduces neuropathic pain and fatigue in cancer patients; EM maintains safer temperatures than TIM	EM: precise temperature control, no smoke/odor, low burn risk. TIM: higher thermal intensity for deep tissue	TIM: burn risk (peak surface temp >50 °C), variable combustion, smoke. EM: limited penetration beyond 5 mm depth.
Combination therapy (acupuncture/EA combined with methylcobalamin or glutathione)	Colorectal cancer; gastrointestinal cancer.	ST36, LI4, SP6, Ashi points	Acupuncture 2–3 times/week + methylcobalamin (84 days).	Synergistic effect: methylcobalamin repairs myelin; acupuncture modulates nerve conduction and blood flow.	↑ Sensory neuropathy response; ↑ NCV; ↓ Pain.	↑ Efficacy vs. monotherapy; ↑ Quality of life; ↑ Sensory symptom relief.	Protocol heterogeneity; requires coordinated drug dosing.
Auricular acupuncture (non-invasive ear point stimulation)	Mixed solid tumors with chronic CIPN	Shenmen, point zero, sympathetic, finger, toe, peripheral nerve, corresponding auricular points	Seeds retained for 3–5 days; replaced weekly; total 3 sessions	Regulates Qi-blood flow in corresponding meridians	• 96% symptom improvement (after 1–2 sessions) • 65% patient satisfaction	• Safe/cost-effective • Rapid onset (1–2 sessions) • No drug interactions	• Mild auricular discomfort/rash • Requires maintenance
ALTENS (transcutaneous electrostimulation, such as LI4, LR3 bilaterally)	Mixed solid tumors with chronic CIPN	/	20 min/session; 2 sessions/week; 10–12 sessions	Modulates pain signaling and nerve function	• 93.8% pain response (59% ↓ VAS) • Sustained mTNS improvement (6 months)	• Non-invasive • Durable effects (6mo+) • Home-applicable (post-training)	• Equipment-dependent • Initial setup complexity

### 3.1 Interventions by modality

#### 3.1.1 Manual acupuncture

Manual acupuncture uses needles at certain points on the body and has shown preliminary benefit for CIPN symptoms. In a pilot randomized sham-controlled trial involving breast cancer survivors with taxane-induced CIPN, manual acupuncture significantly improved tactile perception thresholds, as measured by Semmes–Weinstein monofilaments, and reduced average pain severity (BPI-SF) after 9 weeks of treatment compared to sham acupuncture. Notably, sensory recovery was particularly enhanced at the left-hand middle fingertip and the right-foot big toe tip; however, the small sample limits definitive conclusions (74). D’Alessandro et al. similarly reported within-group improvement in sensory neuropathy, as assessed by the NCI CTCAE scale, but between-group superiority over rehabilitation was not established (75). This pilot study also reported improvements in physical and functional quality-of-life domains. Further evidence from a randomized pilot trial in cancer survivors with moderate-to-severe CIPN indicated that 8 weeks of manual acupuncture reduced neuropathic pain (NRS) and improved CIPN-specific quality-of-life outcomes (FACT/GOG-Ntx) compared to usual care, whereas differences from sham acupuncture were smaller and not consistently significant (13, 14). A systematic synthesis of 33 studies (*n* = 2,027) suggested possible benefit from acupuncture-related interventions (76). These results suggest that manual acupuncture may improve symptoms; however, more research is needed with strong placebo controls to be sure the effects are from acupuncture itself and not just the treatment setting.

#### 3.1.2 Electroacupuncture

Electroacupuncture (EA) uses mild electrical pulses through acupuncture needles. Recent studies have evaluated EA for CIPN. A single-blinded, randomized, sham-controlled pilot trial provides preliminary evidence. In this 60-participant trial, patients with colorectal cancer receiving oxaliplatin underwent 12 weeks of 2-Hz EA and reported lower overall CIPN severity than those receiving sham treatment, with improvements in selected physical and social functioning outcomes; nerve conduction improvement was not established (77). The safety profile was favorable, with adverse events limited to minor hematomas occurring in 0.7% of cases. Notably, the intervention achieved high feasibility, with treatment compliance exceeding 90% during chemotherapy cycles (77). These findings require confirmation in larger trials.

The clinical benefits observed with EA are complemented by mechanistic investigations in animal models. In paclitaxel models, EA modulated Nrf2-related oxidative signaling (40), suppressed TLR4 signaling and TRPV1 upregulation in sensory neurons (73), inhibited spinal glial activation and TLR4/NF-κB signaling (51), and reduced CCL2/CCR2-mediated pro-inflammatory macrophage recruitment (54). In cisplatin models, preventive EA reduced pro-inflammatory microglial activity through neuronal GRK2- and miR-124-dependent mechanisms (52, 53). By contrast, direct CIPN evidence does not currently demonstrate that EA stabilizes microtubules, regulates HDAC6 or Nav channels, or restores mitochondrial biogenesis.

#### 3.1.3 Moxibustion

Moxibustion is a heat therapy that targets acupoints using either burning moxa or electronic heaters. Traditional indirect moxibustion (TIM) uses insulating materials (e.g., ginger) between the moxa cones and the skin, while electronic moxibustion (EM) employs regulated heating, avoiding combustion risks such as burns and smoke (78). Tests on tissue models show that TIM can get too hot, reaching 55 °C, which can damage skin, but EM stays at safer temperatures, about 43 °C, within the range needed for therapy and safe for longer use. The main temperature differences occur near the skin surface (0–2 mm), and EM cools slowly after heating, making it a better option for people who are sensitive to heat or have nerve pain (78).

Moxibustion may help relieve CIPN symptoms such as nerve pain and fatigue. From a biological perspective, thermal stimulation induced by moxibustion may interact with transient receptor potential (TRP) channels, particularly TRPV1, which plays a central role in thermal nociception and inflammatory pain. TRPV1 is frequently sensitized under conditions of chemotherapy-induced oxidative stress and neuroinflammation, contributing to thermal hyperalgesia observed in patients with CIPN. Controlled thermal stimulation generated by moxibustion may activate TRPV1-dependent signaling, subsequently inducing adaptive desensitization of nociceptive pathways and promoting endogenous analgesic responses. This mechanism may partially explain the analgesic effects of moxibustion while highlighting the importance of maintaining therapeutic temperatures within a controlled range. Further studies are required to determine the optimal thermal dose and to clarify how different moxibustion modalities regulate TRPV-related signaling in patients with CIPN (79, 80). Animal studies show that its heat can lower nerve inflammation, possibly by blocking certain pathways (like TLR4/NF-κB). Clinical studies also find that moxibustion can reduce pain and fatigue in cancer patients (81). Electronic moxibustion is especially useful for people with CIPN because it allows you to control the temperature, does not produce smoke or smell, and has a lower risk of skin burns (78, 81). More research is needed to determine the optimal temperatures and treatment times for CIPN, and to examine how moxibustion affects ion channels and mitochondria (78).

#### 3.1.4 Combination therapy

Small trials and meta-analyses suggest that acupuncture combined with pharmacologic agents may improve some CIPN outcomes compared with the pharmacologic comparator alone. Acupuncture with methylcobalamin significantly reduced neuropathic pain (VAS reduction: *p* < 0.01), improved sensory neuropathy symptoms, and increased nerve conduction velocity (sural nerve SCV increase: MD = 3.38 m/s, 95% CI: 1.61–5.15) (82). Electroacupuncture with glutathione (GSH) also produced higher sensory neuropathy response rates than GSH alone (RR = 1.63, 95% CI: 1.08–2.47) (82). Co-treatment with methylcobalamin significantly improved CIPN-specific quality of life (FACT-Ntx: *p* < 0.001) (83). However, heterogeneous protocols and variable study quality limit the certainty of these findings.

Acupuncture combined with standard care reduced pain severity more than standard care alone in pooled analyses (BPI: MD = −1.50, 95% CI: −2.66 to −0.34) (82). Safety profiles were favorable, with no serious adverse events linked to combination regimens (82, 83). Future trials should use standardized protocols and sham-controlled designs to confirm these findings.

### 3.2 Specialized techniques

Specialized acupuncture modalities have shown preliminary findings in refractory CIPN. An uncontrolled case series of 73 patients reported rapid improvement after auricular acupuncture, with 96% reporting improvement after one or two treatments; however, the absence of a comparator and reliance on patient-reported response preclude a causal efficacy estimate (84). ALTENS is a noninvasive procedure in which surface electrodes stimulate acupuncture points. In a prospective phase II study, 27 patients received 12 ALTENS sessions; the median modified Total Neuropathy Score decreased from 7.1 at baseline to 3.1 at 6 months, but pain and well-being results were inconclusive and the study lacked an adequately powered efficacy comparator (85). Separately, the MC5-A Calmare device delivers Scrambler therapy, a patient-specific cutaneous electrostimulation technique that is distinct from ALTENS and acupuncture (86).

### 3.3 Critical appraisal of clinical evidence and remaining challenges

Despite encouraging clinical outcomes, the evidence supporting acupuncture for CIPN remains heterogeneous. Several randomized controlled trials using sham acupuncture or usual-care controls have demonstrated improvements in neuropathic symptoms, sensory function, and quality of life; however, the magnitude of benefit varies considerably among studies. Differences in cancer type, chemotherapy regimen, CIPN severity, acupuncture protocols, control interventions, and outcome measurements contribute substantially to this heterogeneity. Importantly, some sham-controlled trials have reported limited superiority of true acupuncture over placebo procedures, suggesting that nonspecific effects, patient expectation, and contextual factors may contribute to observed improvements (14). A randomized controlled trial investigating acupuncture for prevention of CIPN in colorectal cancer patients receiving chemotherapy found improvements in sensory outcomes and quality-of-life parameters in both true acupuncture and sham acupuncture groups, highlighting the potential contribution of contextual effects, placebo responses, and nonspecific physiological stimulation (87). Therefore, although acupuncture represents a promising supportive intervention, future studies with standardized protocols, validated sham controls, and clinically meaningful effect-size reporting are required.

In summary, studies suggest that manual acupuncture and EA may improve nerve symptoms, reduce pain, and improve quality of life for some patients with CIPN (74–77, 82). Direct preclinical CIPN studies also support selected anti-inflammatory and antioxidant mechanisms (40, 51–54, 73). The 96% response reported for auricular acupuncture came from an uncontrolled case series and should be interpreted as preliminary (84). ALTENS produced sustained improvement in modified Total Neuropathy Scores in a small single-arm phase II study, but pain findings were inconclusive (85). The MC5-A Calmare findings concern Scrambler therapy rather than ALTENS or acupuncture (86). Combining acupuncture with methylcobalamin or glutathione may provide additive benefit, but synergy has not been established (82, 83). Side effects reported in acupuncture studies were generally mild. Current evidence indicates that acupuncture may provide symptomatic benefits for CIPN. But the magnitude of benefit varies among studies, and methodological limitations, including small sample sizes, inconsistent acupuncture protocols, and heterogeneous control designs, prevent definitive conclusions. Therefore, acupuncture should be integrated as a complementary supportive strategy, while further high-quality clinical trials are required to establish standardized and evidence-based treatment protocols.

## 4 Proposed precision-guided acupuncture clinical translation framework for CIPN

### 4.1 Patient stratification: multidimensional individualized strategy

Translating precision acupuncture into effective management of CIPN requires careful patient stratification based on clinical, molecular, and traditional diagnostic criteria. TCM pattern differentiation takes a phenotype-based approach. Cold-type numbness is usually linked to Qi and Yang Deficiency and Blood Stasis, whereas heat-type burning pain is more often seen in Dampness-Heat Obstruction patterns. Proposed links between these patterns and mitochondrial dysfunction or TRP-channel dysregulation have not been validated in CIPN and should be treated as hypotheses rather than treatment-selection rules. Stratification can be further explored by incorporating candidate biomarkers across three main domains. Genetic markers such as SCN9A variants have been associated with CIPN susceptibility in selected cohorts (88). Neurophysiological indices such as QST characterize sensory dysfunction, while slowed nerve conduction velocity indicates large-fiber involvement (89). Circulating biomarkers such as plasma neurofilament light chain may reflect neuroaxonal injury (90). Further clinicopathological profiling considers the chemotherapeutic agents used (e.g., platinum or taxane drugs), the specific neuropathy patterns (sensory-dominant or sensorimotor), and baseline quality-of-life metrics, such as FACT-GOG-Ntx domains (91–94). None of these measures is currently a validated predictor of acupuncture response. Their integration should therefore be evaluated prospectively as a research strategy for addressing CIPN heterogeneity (94, 95).

### 4.2 Hypothesis-generating therapeutic parameter and timing optimization

Treating CIPN with precision acupuncture requires evidence that specific treatment parameters correspond to particular clinical phenotypes or molecular pathways. Direct evidence linking stimulation frequency or acupoint selection to PGC-1α/AMPK, TLR4/NF-κB, Nav, or TRPV regulation in CIPN remains insufficient. A recent randomized trial compared 2, 100, and 2/100 Hz EA with mecobalamin and reported preliminary frequency-related clinical signals; however, it lacked a sham-EA group and did not measure those molecular pathways (96). Thus, proposed mappings of 2 Hz at ST36/GB34 to PGC-1α/AMPK, LI11/SP10 to TLR4/NF-κB, or 100 Hz to Nav/TRPV regulation should be treated as testable hypotheses rather than clinical recommendations. The timing of acupuncture intervention represents another important research question. During active chemotherapy, acupuncture may exert a preventive neuroprotective effect, but available prevention trials remain preliminary and mixed (87, 97). In contrast, patients with established chronic CIPN may require protocols focusing on neuronal repair, sensory recovery, and modulation of central sensitization (98). Future studies should therefore compare prophylactic and rehabilitative timing directly. No direct evidence currently supports administering HDAC6 inhibitors before acupuncture or specifying acupuncture–duloxetine timing around chemotherapy; these concepts are future research directions. Temperature-controlled electronic moxibustion and wearable electrostimulation devices likewise require direct validation in CIPN before they can be recommended for precision treatment.

### 4.3 Clinical validation and implementation pathway

Bringing precision acupuncture into everyday care for CIPN means using practical methods to test what works and to overcome barriers. For clinical testing, a proposed approach is to run trials that include both TCM pattern diagnosis and candidate biomarker tests, comparing these personalized treatments with standard acupuncture. This design could help identify which patients benefit most and sharpen criteria for inclusion. At the same time, real-world registry studies should longitudinally evaluate patient recovery using objective assessments such as quantitative sensory testing (QST), intraepidermal nerve fiber density (IENFD), and validated patient-reported outcome measures. Health economic analyses should further investigate whether acupuncture-based interventions improve chemotherapy adherence, reduce supportive care utilization, and decrease overall healthcare costs. However, robust evidence demonstrating reductions in chemotherapy dose modification or treatment discontinuation remains limited and requires confirmation in large prospective clinical studies (99, 100). Success is not just about pain scores. It is also about whether patients can finish chemotherapy, maintain better balance (Berg Balance Scale), and perform daily activities; whether these outcomes improve alongside FACT-GOG-Ntx scores should be tested prospectively.

## 5 Research gap and future perspectives

Although evidence suggests potential benefit from acupuncture for the management of CIPN, limitations impede clinical translation. One of the main issues is that studies use different methods and do not always follow the same treatment plans or use proper placebo controls. For example, the use of Streitberger needles is not consistent across trials (101). Another problem is measuring the “De Qi” sensation, which is important in acupuncture but is usually reported by patients rather than measured with lab tests (102). Precision medicine cannot move forward easily because patients have not been prospectively stratified by validated mechanism-based CIPN phenotypes. Categories such as “TRPV1-dominant” or “inflammation-dominant” CIPN remain research hypotheses rather than established clinical subgroups (103). Finally, most studies focus on patients’ reports rather than using tests such as skin nerve fiber counts or sensory testing, so results are not as complete as they could be (104).

To close these gaps, future precision-acupuncture studies may combine clinical phenotype with candidate measures such as SCN9A genotype, neurofilament light chain, and quantitative sensory testing (88–90). These variables should first be evaluated as predictors or effect modifiers and should not yet be used routinely to select patients. Neuroimaging may also serve as an exploratory outcome. Acupuncture at GV20 and GV29 altered default-mode-network connectivity in 30 healthy participants, but this study did not include patients with CIPN and does not validate fMRI-guided acupoint selection (105). Wearable electrostimulation with real-time feedback is similarly a future research objective rather than an established CIPN intervention (104).

## 6 Conclusion

Acupuncture is a promising adjunctive approach for preventing and treating CIPN. Although current clinical evidence suggests that it may alleviate neuropathic pain, improve sensory symptoms, and enhance quality of life, sham-controlled findings are not uniformly positive. Direct animal studies support modulation of selected pathways, including Nrf2-related oxidative signaling, TLR4/TRPV1 signaling, spinal glial activation, neuronal GRK2 and miR-124, and CCL2/CCR2-mediated macrophage recruitment (40, 51–54, 73). By contrast, acupuncture-mediated microtubule stabilization, HDAC6 regulation, mitochondrial biogenesis, and Nav/Kv/Cav-channel normalization remain unconfirmed in CIPN. Inconsistent methodological rigor in clinical trials, unresolved quantification of “De Qi,” inadequate patient stratification, and overreliance on subjective endpoints hinder robust clinical translation. To bridge these gaps, the proposed precision acupuncture framework integrating TCM pattern differentiation with molecular profiling and neuroimaging should be viewed as a research agenda requiring prospective validation. Wearable devices likewise require direct CIPN evaluation before clinical implementation. Future research should focus on trials that use clear standards and include objective outcomes and candidate biomarkers. Beyond symptom relief, acupuncture offers several potential clinical advantages for patients with CIPN. Current evidence suggests that acupuncture is a safe, well-tolerated complementary therapy that may improve neuropathic pain, sensory function, quality of life, and functional recovery. Although the overall certainty of current evidence remains limited by methodological heterogeneity and the need for larger sham-controlled trials, its favorable safety profile supports acupuncture as a promising adjunctive strategy within multidisciplinary CIPN management. Future trials should determine which patients benefit most and whether acupuncture affects chemotherapy completion or long-term outcomes.
